# On the Utility of Short Echo Time (TE) Single Voxel 1H–MRS in Non–Invasive Detection of 2–Hydroxyglutarate (2HG); Challenges and Potential Improvement Illustrated with Animal Models Using MRUI and LCModel

**DOI:** 10.1371/journal.pone.0147794

**Published:** 2016-01-28

**Authors:** Hwon Heo, Sungjin Kim, Hyeong Hun Lee, Hye Rim Cho, Wen Jun Xu, Se-Hoon Lee, Chul-Kee Park, Sunghyouk Park, Seung Hong Choi, Hyeonjin Kim

**Affiliations:** 1 Department of Biomedical Sciences, Seoul National University, Seoul, Korea; 2 Department of Radiology, Seoul National University Hospital, Seoul, Korea; 3 College of Pharmacy, Natural Product Research Institute, Seoul National University, Seoul, Korea; 4 Department of Internal Medicine, Seoul National University Hospital, Seoul, Korea; 5 Department of Neurosurgery, Seoul National University Hospital, Seoul, Korea; 6 Institute of Radiation Medicine, Seoul National University Medical Research Center, Seoul, Korea; Mayo Clinic, UNITED STATES

## Abstract

Mutations in isocitrate dehydrogenase 1 and 2 (IDH1/2) are frequently found in brain tumors, and the resulting onco–metabolite, 2–hydroxyglutarate (2HG), has been suggested to be a potential diagnostic and prognostic biomarker of the diseases. Indeed, recent studies have demonstrated the feasibility of non–invasively detecting 2HG by using proton magnetic resonance spectroscopy (1H–MRS). Due to severe spectral overlaps of 2HG with its background metabolites and spectral baselines, however, the majority of those previous studies employed spectral editing methods with long echo times (TEs) instead of the most commonly used short TE approach with spectral fitting. Consequently, the results obtained with spectral editing methods may potentially be prone to errors resulting from substantial signal loss due to relaxation. Given that the spectral region where the main signal of 2HG resides is particularly sensitive to spectral baseline in metabolite quantification, we have investigated the impact of incorporating voxel–specifically measured baselines into the spectral basis set on the performance of the conventional short TE approach in 2HG detection in rodent models (Fisher 344 rats; n = 19) of IDH1/2 mutant–overexpressing F98 glioma at 9.4T. Metabolite spectra were acquired (SPECIAL sequence) for a tumor region and the contralateral normal region of the brain for each animal. For the estimation of spectral baselines metabolite–nulled spectra were obtained (double–inversion–recovery SPECIAL sequence) for each individual voxels. Data were post–processed with and without the measured baselines using MRUI and LCModel—the two most widely used data post–processing packages. Our results demonstrate that *in–vivo* detection of 2HG using the conventional short TE approach is challenging even at 9.4T. However, incorporation of voxel–specifically measured spectral baselines may potentially improve its performance. Upon more thorough validation in a larger number of animals and more importantly in human patients, the potential utility of the proposed short TE acquisition with voxel–specific baseline measurement approach in 2HG detection may need to be considered in the study design.

## Introduction

Glioblastomas and malignant gliomas are the common phenotypes of brain tumors. They are the most aggressive diffuse gliomas of astrocytic lineage [1, 2] with poor prognosis and lower survival. According to the World Health Organization (WHO) Classification of Tumors of the Central Nervous System, mutations in isocitrate dehydrogenase 1 and 2 (IDH1/2) are found in grade II and III astrocytomas and oligodendrogliomas, and in grade IV glioblastomas [3, 4]. IDH is a nicotinamide adenine dinucleotide phosphate (NADP+)–dependent enzyme, which normally catalyzes the oxidative decarboxylation of isocitrate to α–ketoglutarate (α–KG). Most of IDH1 mutations occur at a single amino acid residues, R132 (predominantly R132H), in the active site of the enzyme [4, 5]. For IDH2 mutations, the disease–associated single residual mutations are highly present at the R172 residue (predominantly R172K) [4, 6]. These IDH1/2 mutants acquire a new enzymatic ability to catalyze the NADPH–dependent reduction of α–KG to 2–hydroxyglutarate (2HG) [7]. Because it is an error product of abnormal metabolism in brain tumors with IDH mutation, 2HG is considered an onco–metabolite [8]. Together with the implication of the IDH mutational status with patient survival duration [4], therefore, developing a noninvasive means of detecting 2HG would have a great diagnostic and prognostic value [9–11].

Proton magnetic resonance spectroscopy (1H–MRS) has been extensively used for quantification of metabolites in vivo. Typically in these days, 1H–MRS spectra are acquired at the shortest echo time (TE) attainable in order to minimize signal loss resulting from relaxation and, for coupled spins, J–evolution. Then, spectral fitting is performed for quantification of individual metabolites. Indeed, recent 1H–MRS studies have clearly demonstrated its potential ability to non–invasively detect the presence of 2HG in glioma patients with IDH mutations [9, 10, 12]. However, due to the severe spectral overlaps of 2HG signal with its background metabolite signals (e.g., gamma–aminobutylic acid (GABA), glutamate (Glu), glutamine (Gln), and N–acetylaspartylglutamate (NAAG)) [9, 10], the majority of those previous studies [9, 10] had to employ spectral editing methods [13] with a long TE indispensably where those background metabolite signals are either effectively suppressed by taking advantage of different J–evolution of different coupled spin systems [10] or avoided by J–difference editing [9, 10]. Thus, the resulting 2HG concentrations are prone to quantitative errors due to substantial signal loss over such a long TE as previously discussed [10, 14]. The quantification of metabolites other than 2HG can also be challenging in the edited spectra due to the same signal loss mechanisms, which may also be important for better understanding of the pathogenesis and progression of brain tumors with gene mutations [15–17]. Thus, the conventional short TE approach is still desired despite its reported limitation in the detection of 2HG [9, 12, 14].

Previous studies have demonstrated the strong dependence of the accuracy of metabolite quantification on the characteristics of spectral baseline [18–20] that is mainly contributed by macromolecules (MMs) at short TE [21]. In particular, quantification of Glu and Gln is known to be most influenced by baseline [18, 19] and the difficulty of separating Glu and Gln had been attributed more to baseline than to their spectral overlaps [22]. Therefore, given that the main signal of 2HG directly overlaps with both Glu and Gln [9, 10], and that measured baselines provide more information than those modelled [23], potential impact of incorporating measured baselines into the spectral basis set on the detection of 2HG at short TE should be an important issue, which has not been addressed in the previous 1H–MRS 2HG studies [9, 10, 12, 14]. It should also be noted that all those previous studies used LCModel [22] for metabolite quantification. While LCModel is most commonly used for 1H–MRS data post–processing in frequency–domain, MRUI [24] is another frequently used software package [11] employing time–domain analysis. While the overall performance of such frequency– or time–domain spectral analyses were reported to be comparable on the one hand [25–27], they are also known to have their own characteristics [27] on the other hand.

To this end, we have assessed the performance of the conventional short TE 1H–MRS in the detection of 2HG in a rodent model of IDH1/2 mutant–overexpressing F98 glioma at 9.4T. Spectral baselines were obtained for each individual voxels, and data were post–processed by using both MRUI and LCModel with and without the incorporation of the measured baselines into the spectral basis set [18, 20]. Based on the results, technical challenges and potential strategies are discussed for the improvement of the efficacy of the short TE approach in the detection of 2HG in vivo.

## Materials and Methods

The animal research protocol was approved by the Institutional Animal Care and Use Committee (IACUC) of Seoul National University Hospital. Prior to MR data acquisition, rats were anesthetized in a chamber with isoflurane (1.5% in oxygen). Rats were anesthetized with 20mg/kg of Zoletil^®^ 100 (tiletamine–zolazepam, Virbac, Carros, France) for glioma implantation. Rats were euthanized by using CO_2_.

### F98 glioma culture

Rat F98 glioma cells were obtained from the American Type Culture Collection (ATCC, Rockville, MD, USA) and were grown as previously described [28]. Briefly, F98 cells were maintained in Dulbecco’s modified Eagle medium (DMEM) with 10% fetal bovine serum (FBS) at 37°C. To express IDH1 wild–type (IDH1–WT), IDH1–R132H, IDH2 wild–type (IDH2–WT), IDH2–R172K, and Mock transgenes, F98 cells were transfected with lentivirus for 24 h in the presence of 4–8 μg/mL polybrene. Transfected cells were passed for no more than 10 passages. These cells were maintained in DMEM including 10% FBS and penicillin (Hyclone, Logan, UT, USA). The cells were cultured at 37°C and 5% CO_2_ in a 90% humidified incubator.

### Construction and preparation of lentiviral vectors

The IDH1/2 lentiviral vectors were prepared as previously described [29]. Specifically, the genes for human IDH1 (GenBank accession number NM_005896) and IDH2 (GenBank accession number NM_002168) were obtained from Origene (Rockville, MD, USA), PCR amplified, and cloned into lentiviral vector CD526A–1 (System Biosciences, Mountain View, CA, USA). IDH1–R132H and IDH2–R172K were amplified with the above clones as templates using standard site directed mutagenesis. The cloned inserts were expressed under enhanced constitutive suCMV promoter. The sequences of the cloned genes were confirmed by ABI BigDye^®^ Terminator Cycle Sequencing Kit (Foster city, CA, USA). The recombinant lentivirus was produced by SeouLin Bioscience Institute (Daejon, Korea). Expression of lentiviral particles was produced in HEK–293T cells cultured in DMEM including 10% FBS and collected after 48 h. Then, the virus was filtered through 0.45 μm membrane filter (Millipore, Billerica, MA, USA), and immediately stored at -70°C. Titer was determined by the TCID50 method and the concentrated titer was 2×10^7^ IFU/mL.

### Immunoblot analysis

The protein levels of F98 IDH1 –WT/–R132H, IDH2 –WT/–R172K, and Mock gliomas were evaluated by immunoblot analysis. Cells were lysed in ice–cold lysis buffer composed of 20 mM Tris–HCl (pH 7.5), 150 mM NaCl, 1 mM Na_2_EDTA, 1 mM EGTA, 1% Triton, 2.5 mM sodium pyrophosphate, 1 mM β–glycerophosphate, 1 mM Na_3_VO_4_, 1 μg/mL leupeptin, and protease inhibitor cocktail (Sigma, MO, USA), and the concentration of lysate protein was evaluated with the bicinchoninic acid method (Pierce Biotechnology, Rockford, IL, USA). Approximately 50 μg of protein was loaded in each lane of a polyacrylamide denaturing gel for electrophoresis. After electrophoresis, the protein was transferred to nitrocellulose membranes for blotting. We used a rat monoclonal antibody to IDH1 (Dianova, Hamburg, Germany), a mouse monoclonal antibody to IDH1–R132H (Dianova), a rabbit polyclonal antibody to IDH2 (Proteintech, Chicago, IL, USA), a mouse monoclonal antibody to IDH2–R172K (NewEast Biosciences, King of Prussia, PA, USA), and a rabbit polyclonal antibody to β–actin (Abcam, Cambridge, UK). Primary antibodies were detected by horseradish peroxidase–conjugated antibodies (Santa Cruz Biotechnology, Paso Pobles, CA, USA).

### F98 rat glioma implantation

The animal research protocol was approved by the Institutional Animal Care and Use Committee (IACUC). Six–week–old Fisher 344 female rats (F98 IDH1–WT, n = 3; F98 IDH1–R132H, n = 3; F98 IDH2–WT, n = 3; F98 IDH2–R172K, n = 10) with orthotopic brain tumors were used. Rats were anesthetized with 20mg/kg of Zoletil^®^ 100 (tiletamine–zolazepam, Virbac, Carros, France). Rats were mounted on a stereotactic frame. A midline scalp incision was performed, followed by identification and exposure of the bregma. For induction of the brain tumors, stable gene transferred F98 cells were transplanted into the right striatum by using a 10 μL Hamilton syringe (AP, 1.0 mm; ML, 3.0 mm; DV, 5.5 mm). The needle was then slowly withdrawn. Rats were given a continuous one–layer suture, and post–care was performed in the cage.

### MR data collection

In vivo 1H–MRS was performed at 2 weeks after the implantation of F98 glioma cells. Prior to data acquisition, rats were anesthetized in a chamber with isoflurane (1.5% in oxygen), and placed in the magnet in the prone position. During data acquisition anesthesia was maintained (1.0–1.5% isoflurane in oxygen) and the respiration and body temperature of the animals were monitored.

All MR data were collected on a 9.4T MR scanner (Agilent 9.4T/160AS; Agilent Technologies, Santa Clara, CA, USA) using a single channel surface coil (20 mm in diameter; Agilent Technologies) for both radio–frequency (RF) transmission and signal reception. Scout images were acquired by using a gradient echo sequence for all three orthogonal directions (repetition time (TR)/echo time (TE) = 55/2.75 ms, field–of–view (FOV) = 50×50 mm^2^, matrix size = 128×128, 10 slices for each direction (no gap), slice thickness (TH) = 1 mm, receiver bandwidth (BW) = 50 kHz, 1 signal average). Additional anatomical images were acquired in the axial direction using a T2–weighted fast spin echo sequence (TR/TE = 3000/30 ms, echo train length (ETL) = 4, FOV = 35×35 mm^2^, matrix size = 192×192, number of slices = 15 (no gap), TH = 1 mm, receiver BW = 100 kHz, 2 signal averages).

Based on the anatomical images, 1H–MRS voxels were defined in a tumor region (tumor VOI) and the contralateral normal region (CN VOI) of the brain for each animal. The voxel volumes were slightly adjusted depending on the tumor size, and the 1^st^–and 2^nd^–order shimming was performed over the voxels. 1H–MRS data were acquired with a SPECIAL sequence [30] (TR/TE = 4000/2.83 ms, spectral BW = 5 kHz, 2048 data points, 384 signal averages). A 0.6 ms (BW = 4.4 kHz) slice–selective Gaussian pulse was used for excitation. For inversion and refocusing a 3 ms (BW = 6.6 kHz) and a 1.3 ms (BW = 15.6 kHz) hyperbolic secant adiabatic full passage (AFP) pulses were used, respectively. To minimize voxel displacement [31], the carrier frequencies of the RF pulses of the SPECIAL sequence were adjusted by -2.3 ppm from the water resonance. A 32–step phase cycling was employed. Both a VAPOR water suppression [32] and an outer volume suppression (OVS) modules were also used [33].

For the estimation of spectral baselines, metabolite–nulled spectra [21] were also acquired for all voxels using a double inversion [23, 34, 35] SPECIAL sequence with nonselective hyperbolic secant pulses for inversion (duration = 3 ms and BW = 6.6 kHz). The first and the second inversion times (TI_1_ and TI_2_, respectively) were optimized according to the previous reports [23, 34, 35]. Briefly, a TI_2_ was determined first in consideration of the duration of the VAPOR and OVS modules. Then, using the T1’s of metabolites reported previously at 9.4T [36], a TI_1_ for metabolite nulling was obtained for each individual metabolites [34, 35], from which a compromised, optimal TI_1_ was determined [35]. The sequence parameters for the optimized double inversion SPECIAL sequence were; TI_1_/TI_2_/TR = 2830/680/4650 ms, 320 signal averages. The rest of the sequence parameters were identical to those used for the metabolite quantification. Metabolite–nulled spectra at a long TE (TE = 30 ms, 160 signal averages) were also acquired to examine residual metabolite signals and validate optimal inversion parameters (data not shown).

Water–unsuppressed spectra were also collected from all voxels for the estimation of water content, and as a measure of SNR and linewidth of the spectra (32 signal averages).

### Brain sample

Following the MRS scan, rats were sacrificed, and brain tissues were collected for each VOI, and frozen on dry ice. The tissues were then stored at -80°C.

### Liquid chromatography–mass spectrometry (LC–MS)

The 2HG levels were measured using liquid chromatography–mass spectrometry (LC–MS). All tumor VOI samples (IDH1/2–WT, n = 6; IDH1/2–MT, n = 13) and six of the CN VOI samples were analyzed. Specifically, the frozen tissues were weighed, thawed at room temperature and homogenized with 300 μL mixture composed of methanol, acetonitrile, and distilled water (5:3:2). The samples were centrifuged at 28,000 g for 30 minutes, and the supernatants were used as the analytes. Quantitative analyses were performed by using an Agilent 1100 Series liquid chromatography system (Agilent, CO, USA). Sample separation was achieved by injecting 2 μL samples into a ZIC–pHILIC polymeric beads peek column (150×2.1 mm, 5 μm, Merck, Germany) at 35°C with a 0.15 mL/min flow rate. 10 mM ammonium carbonate (pH = 8.9) in distilled water was used as mobile phase A, and acetonitrile (ACN) as mobile phase B. The linear gradient was used as follows: 80% B at 0 minute, 35% B at 10 minutes, 5% B at 12 minutes, 5% B at 25 minutes, 80% B at 25.1 minutes, and 80% B at 35 minutes. Mass spectra were obtained in negative ion mode using an API 2000 Mass Spectrometer (AB/SCIEX, Framingham, MA, USA). The ESI source operation parameters were: -4.5 kV of ion spray and the heater (turbo) gas temperature at 350°C. Multiple reaction monitoring (MRM) was performed and controlled by Analyst 1.6 Software.

### 1H–MRS data analysis by MRUI and LCModel

#### Processing of metabolite–nulled spectra

The metabolite–nulled spectra acquired for each individual voxels were analyzed by MRUI (v. 5.0). Given the low signal yield of spectral baseline in the metabolite–nulled spectra with respect to that in the metabolite spectra resulting from double inversion [36] as well as from the lower number of signal averages (320 vs. 384), the metabolite–nulled spectra were apodized [37] such that the SNR of the MM resonance at ~0.9 ppm therein was comparable to that in the metabolite spectra. Then, HLSVD filter [38] was applied to remove unwanted signal.

#### Metabolite quantification using MRUI after FID–truncation (FID–truncation+MRUI)

To investigate the impact of incorporating measured baseline into the spectral fitting on 2HG detection by using MRUI, first, initial data points of the free induction decays (FIDs) of the metabolite spectra were truncated for the removal of MM signal [23, 35, 39, 40]. The number of data points to be truncated was determined empirically [23, 35, 41]. That is, the SNR of the total creatine (tCr; ~3.0 ppm) and N–acetylaspartate (NAA; ~2.0 ppm) peaks and the reduction of MM signal were examined for all spectra by varying the number of truncated FID points from 1 to 30. Then, an optimal number of truncated FID points was determined to be 12.

The spectral bases for a total of 19 metabolites were created by using GAMMA [42] by referring to the previously reported chemical shifts and J–coupling constants of the metabolites [10, 43]. As a routine, data were zero–filled to 4096 points and, after Fourier transformation, line–broadened (~7 Hz) and phase–corrected. Residual water signal was removed by the HLSVD filter. Finally, individual metabolites were quantified by using QUEST [44].

#### Metabolite quantification using MRUI with measured baseline (voxel–specific baseline+MRUI)

The preprocessed metabolite–nulled spectra were included in the spectral basis set voxel–specifically, followed by the routine procedure for metabolite quantification.

#### Metabolite quantification using LCModel with simulated (built–in) baseline (simulated baseline+LCModel)

1H–MRS data were processed by using LCModel (v. 6.3–1J) with the same metabolite basis set that was used with MRUI. The built–in, vendor–provided, modeled MM and lipid components were included in the spectral fitting.

#### Metabolite quantification using LCModel with measured baseline (voxel–specific baseline+LCModel)

The preprocessed metabolite–nulled spectra were included in the spectral basis set voxel–specifically. The built–in, modelled MM and lipid components were excluded in the fitting process in order to minimize over–parameterization of the analysis [45], while retaining the spline function.

#### Processing of water–unsuppressed spectra

The water–unsuppressed data were processed by using MRUI with the routine procedure. The SNR of water signal was estimated by dividing the peak amplitude by the standard deviation of noise measured in the 8–10 ppm range. The linewidth of water signal was estimated from the full–width–at–half–maximum.

The 2HG content estimated by 1H–MRS was normalized to that of water (to be denoted as 2HG_MRS_ wherever in need of distinction from the 2HG content estimated by LC–MS (2HG_LC–MS_)).

#### Combined data analysis

First, the voxels from F98 IDH1/2 WT/MT and CN VOI were stratified into Group A and Group B according to their 2HG content (relative intensity (RI)) measured by LC–MS (2HG_LC–MS_). The Group A and Group B were then considered as 2HG–absent and 2HG–present, respectively. Given the limited SNR and spectral dispersion of 1H–MRS spectra, those samples with intermediate 2HG_LC–MS_ were excluded in the final data analysis. A Cramer–Rao lower bound (CRLB) of less than 20% [10, 14, 46] was considered as indicating successful spectral fitting. According to the 2HG_MRS_ and associated CRLBs, the detection outcome with 1H–MRS was classified into true positive (Tp), false positive (Fp), true negative (Tn), false negative (Fn), and uncertain (U; CRLB>20%) cases.

To address those uncertain cases with CRLB>20%, as an illustration, the relationships between 2HG_LC–MS_ and 2HG_MRS_ were obtained by linear regression for each of the voxel–specific baseline+MRUI and the voxel–specific baseline+LCModel data including only those MRS data with correct detection. Using these relationships, the highest 2HG_LC–MS_ in Group A (2HG–absent) was converted into 2HG_MRS_ values, which were then defined as the cutoff 2HG_MRS_ values for 2HG–absent voxels. That is, those voxels with CRLB>20% were considered either 2HG–absent or 2HG–present [12, 14] according to these cutoff 2HG_MRS_ values in this study.

## Results

All results are expressed in mean±standard deviation (SD).

### Establishment of F98 glioma cell lines for IDH –WT and –R132H/–R172K expression

**Fig 1:**
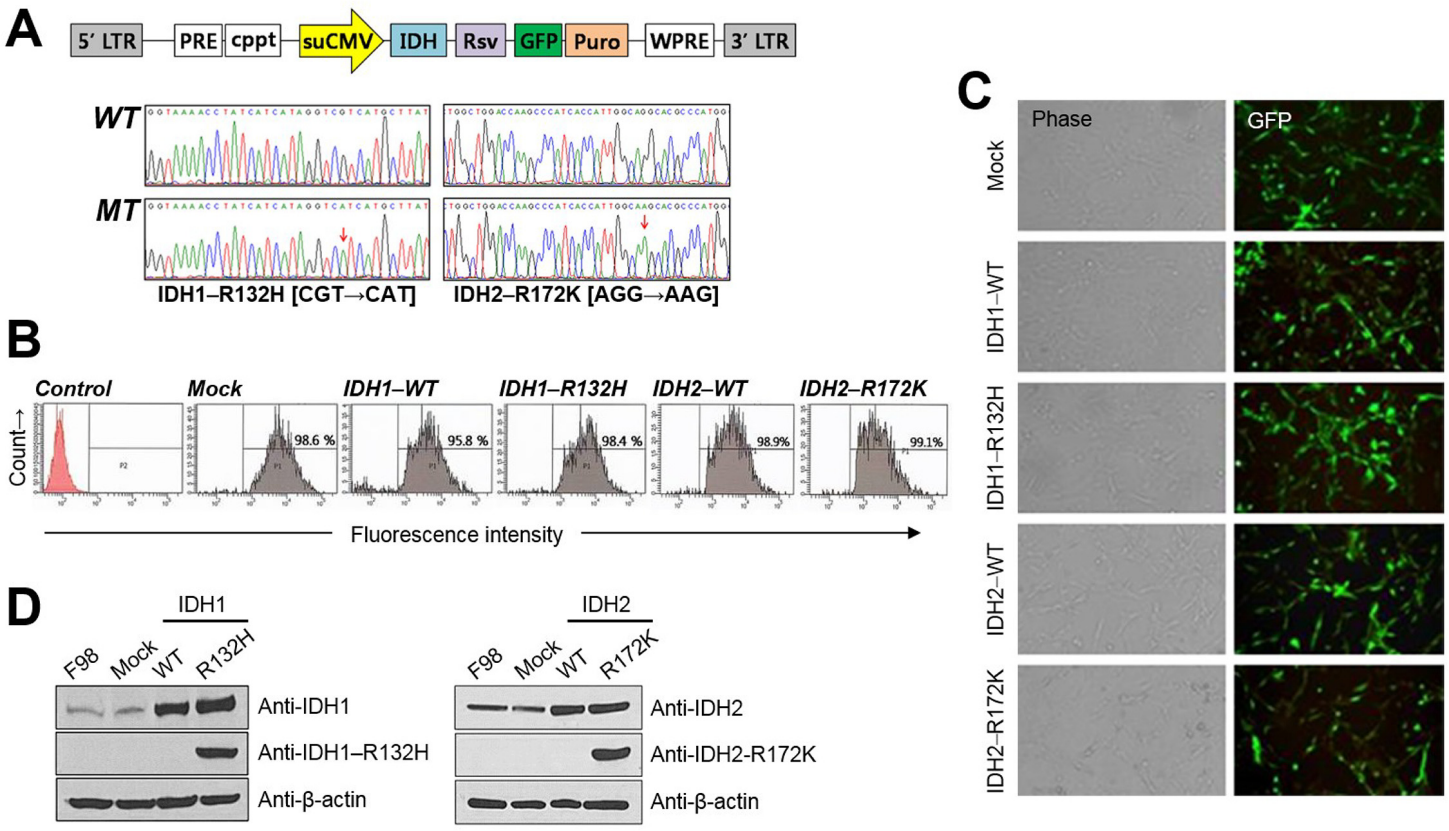
Establishment of IDH1–wild type (WT), IDH1–mutant (–R132H), IDH2–WT, and IDH2–mutant (–R172K) overexpressing F98 cell lines. (A) Construction map of IDH1/2–WT or –R132H/–R172K lentiviral vector. (B) GFP expressions in Mock, IDH1/2–WT or –R132H/–R172K vector transduced F98 cells by using Fluorescence–activated cell sorting (FACS). (C) GFP–tagged gene expressions of the F98 cells confirmed by fluorescent microscopic images. (D) Immunoblot analysis where IDH1–R132H or IDH2–R172K specific antibodies were detected only in the mutated epitopes of F98 IDH1–R132H or IDH2–R172K, respectively.

After gene transfer into F98 glioma cell lines with lentiviral vectors containing IDH1/2–WT–GFP, IDH–R132H/–R172K–GFP, or empty–mock–GFP expression codons (Fig 1A), GFP–expressing F98 cells represented ~95.8–99.1% of the total population (Fig 1B). The fluorescent microscopic images (Fig 1C) and the immunoblot analysis (Fig 1D) further confirmed the well–established F98 glioma cell lines for the IDH1/2–WT and IDH–R132H/–R172K expressions.

### 2HG levels in the samples as measured by LC–MS (2HG_LC–MS_)

**Fig 2:**
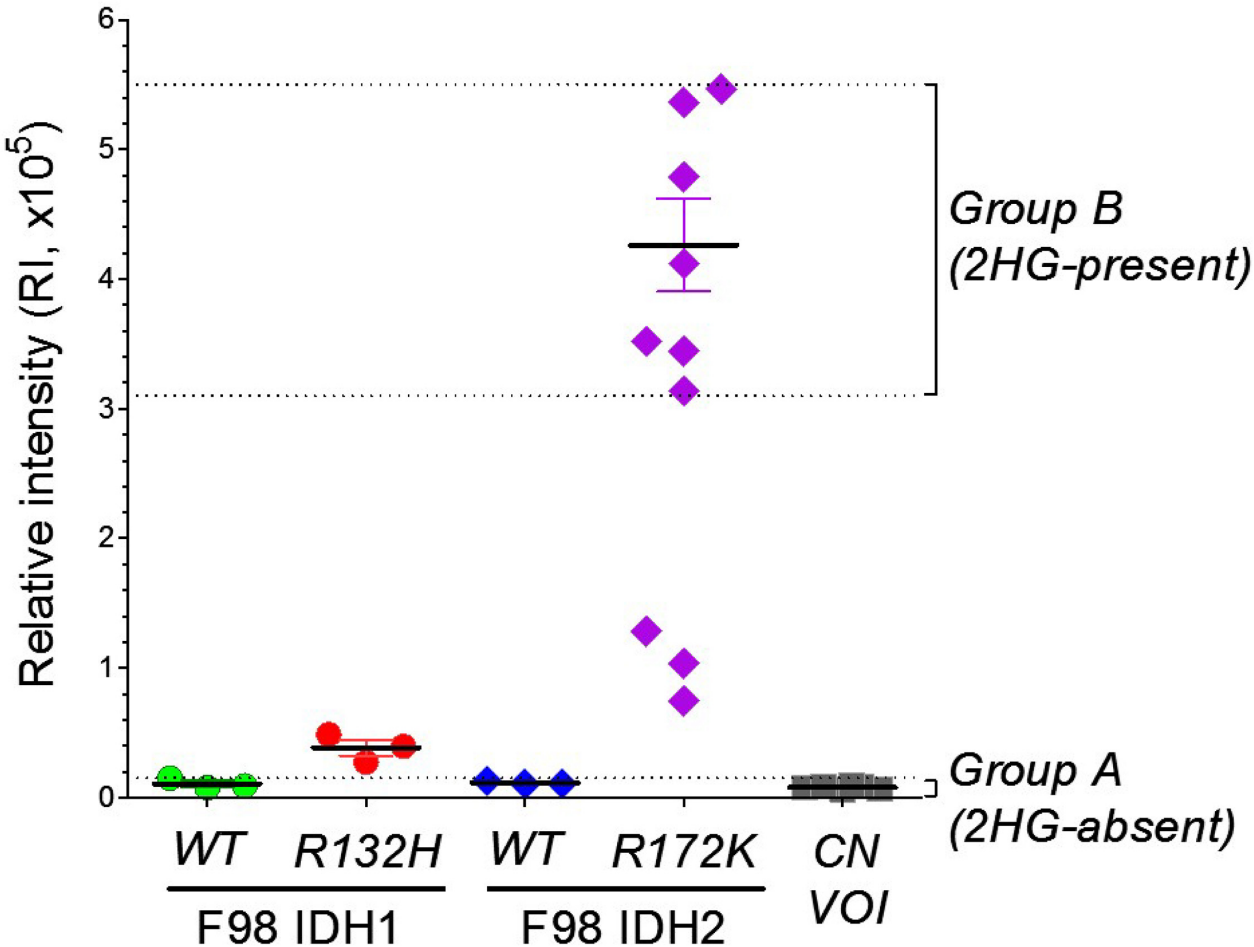
2–hydroxyglutarate (2HG) levels in the brain samples as measured by *ex vivo* liquid chromatography–mass spectrometry (LC–MS). The brain samples were collected from the tumor regions (F98 IDH1/2–WT, IDH1–R132H, and IDH2–R172K) and the contralateral, normal regions (CN VOI). The relative intensity of 2HG in Group A (n = 12) and Group B (n = 7) ranged 0.06x10^5^~0.15x10^5^ and 3.14x10^5^~5.47x10^5^, respectively. Those 6 samples with intermediate 2HG levels (3 from IDH1–R132H and 3 from IDH2–R172K) were excluded in the final data analysis, and then the Group A and Group B were treated as 2HG–absent and 2HG–present, respectively.

The 2HG_LC–MS_ values in the brain samples are shown in Fig 2. Based on these 2HG_LC–MS_ values, samples were classified into Group A (n = 12; 2HG–absent) and Group B (n = 7; 2HG–present). Given the limited SNR and spectral dispersion of 1H–MRS, those 6 samples with intermediate 2HG_LC–MS_ were excluded from the further analysis. The lowest 2HG_LC–MS_ in Group B was more than 20 times higher than the highest 2HG_LC–MS_ in Group A.

### 1H–MRS spectra

**Fig 3:**
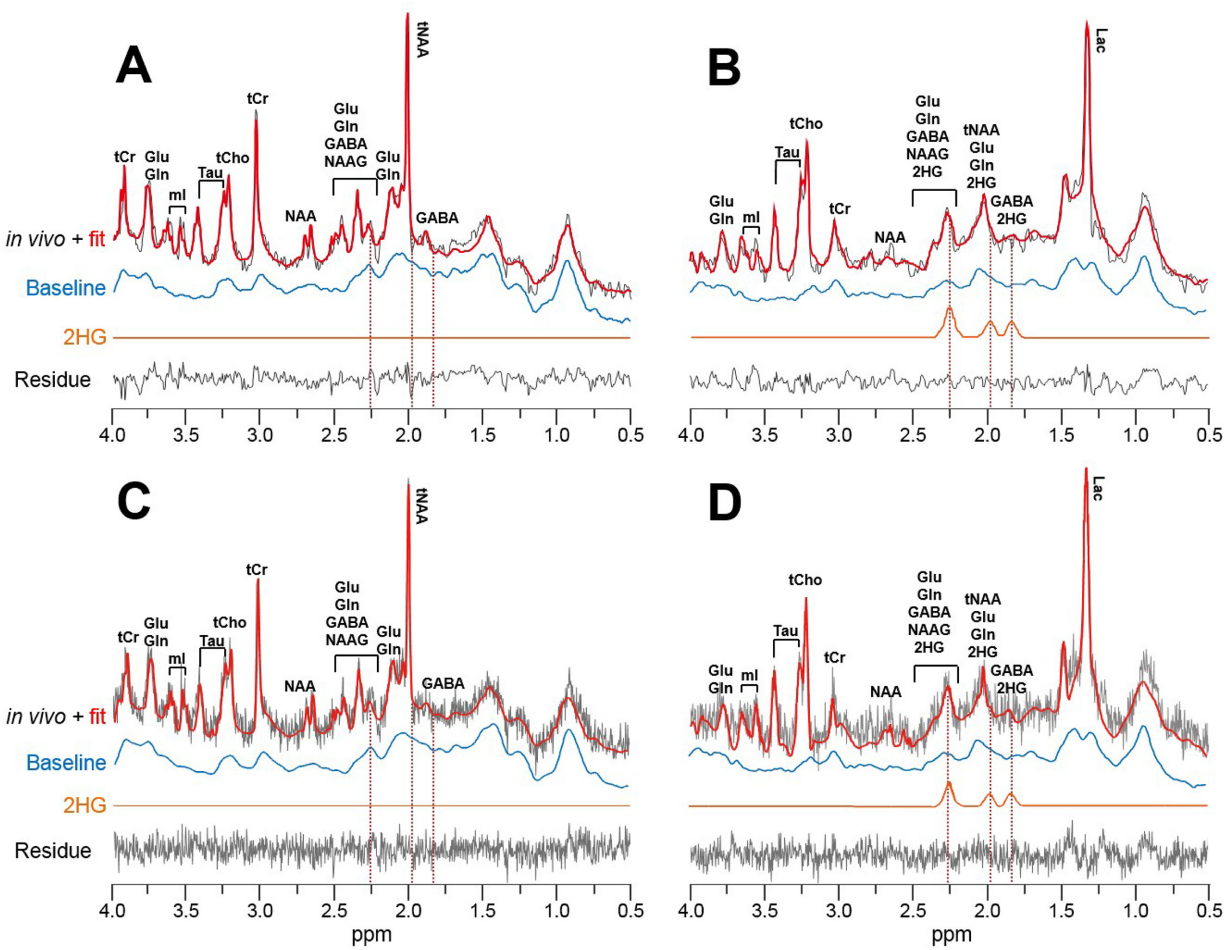
Representative 1H–MRS spectra. (A and C) A contralateral normal brain region (CN VOI). (B and D) A brain tumor region (tumor VOI) with F98 IDH2–R172K glioma. All spectra were post–processed with voxel–specifically obtained spectral baselines by using either MRUI (A and B) or LCModel (C and D). The resulting residual of fit and the 2HG spectral components are also shown, which were denoted by dashed lines in ~1.8–2.3 ppm. (2HG: 2–hydroxyglutarate, GABA: gamma–aminobutylic acid, Gln: glutamine, Glu: glutamate, Lac: lactate, mI: myo–inositol, NAA: N–acetylaspartate, NAAG: N–acetylaspartylglutamate, Tau: taurine, tCho: total choline, tCr: total creatine, tNAA: total N–acetylaspartate).

Representative 1H–MRS spectra are shown in Fig 3 for a CN VOI (Fig 3A and 3C) and a tumor VOI (Fig 3B and 3D), which were post–processed by either MRUI (Fig 3A and 3B) or LCModel (Fig 3C and 3D) with the voxel–specifically measured spectral baselines. The baselines for CN VOI are in good agreement with those previously reported at the same field strength [20].

The mean linewidth and SNR of water peaks in the water–unsuppressed spectra were 15.05±3.31 Hz and 1.72×10^3^±4.06×10^2^, respectively, for the mean voxel volume of 9.58±1.73 mm^3^.

### Impact of voxel–specific baseline on 2HG detection by 1H–MRS

The results of 2HG detection by 1H–MRS are summarized in Table 1.

**Table 1:** The results of 2HG detection by 1H–MRS. T_n_, true-negative; T_p_, true-positive; F_n_, false-negative; F_p_, false-positive; U, uncertain case (CRLB >20%); n/a, not available;

	MRUI							LCModel						
*Rat number (tissue type)*	*FID Truncation*			*Voxel-specific baseline*				*Simulated baseline*			*Voxel-specific baseline*			
	*2HG /water*	*2HG CRLB(%)*	*Detectability*	*2HG /water*	*2HG CRLB(%)*	*Detectability*	Detectability (2HG cut-off* *consideration)*	*2HG /water*	*2HG CRLB(%)*	*Detectability*	*2HG /water*	*2HG CRLB(%)*	*Detectability*	*Detectability (2HG cut-off*^#^ *consideration)*
*Group A (2HG-absent)*														
#1 (F98 IDH1-WT)	0.00	n/a	T_n_	0.00	n/a	T_n_	T_n_	2.83	20	F_p_	1.69	47	U	F_p_
#2 (F98 IDH1-WT)	0.00	n/a	T_n_	0.67	31	U	F_p_	3.32	15	F_p_	0.28	217	U	T_n_
#3 (F98 IDH1-WT)	0.00	n/a	T_n_	0.03	424	U	T_n_	2.89	21	U	0.77	104	U	F_p_
#4 (F98 IDH2-WT)	0.00	n/a	T_n_	0.27	54	U	F_p_	2.22	18	F_p_	0.00	999	T_n_	T_n_
#5 (F98 IDH2-WT)	1.37	21	U	0.00	n/a	T_n_	T_n_	3.28	13	F_p_	1.46	36	U	F_p_
#6 (F98 IDH2-WT)	0.70	85	U	0.00	n/a	T_n_	T_n_	4.50	17	F_p_	0.00	999	T_n_	T_n_
#7 (CN VOI)	0.00	n/a	T_n_	0.00	n/a	T_n_	T_n_	2.82	20	F_p_	0.76	74	U	F_p_
#8 (CN VOI)	0.00	n/a	T_n_	0.00	n/a	T_n_	T_n_	2.69	20	F_p_	0.28	217	U	T_n_
#9 (CN VOI)	0.00	n/a	T_n_	0.00	n/a	T_n_	T_n_	3.28	18	F_p_	0.86	83	U	F_p_
#10 (CN VOI)	0.00	n/a	T_n_	0.00	n/a	T_n_	T_n_	2.72	23	U	0.00	999	T_n_	T_n_
#11 (CN VOI)	0.00	n/a	T_n_	0.00	n/a	T_n_	T_n_	2.04	24	U	0.00	999	T_n_	T_n_
#12 (CN VOI)	0.00	n/a	T_n_	0.00	n/a	T_n_	T_n_	3.05	21	U	0.80	100	U	F_p_
*Group B (2HG-present)*														
#13 (F98 IDH2-R172K)	0.03	447	U	5.52	7	T_p_	T_p_	4.12	8	T_p_	5.31	15	T_p_	T_p_
#14 (F98 IDH2-R172K)	2.30	10	T_p_	2.91	7	T_p_	T_p_	6.80	11	T_p_	4.42	24	U	T_p_
#15 (F98 IDH2-R172K)	4.09	6	T_p_	2.37	9	T_p_	T_p_	3.83	9	T_p_	3.71	13	T_p_	T_p_
#16 (F98 IDH2-R172K)	0.00	n/a	F_n_	0.48	54	U	T_p_	5.75	13	T_p_	6.76	19	T_p_	T_p_
#17 (F98 IDH2-R172K)	3.97	5	T_p_	8.64	5	T_p_	T_p_	7.49	10	T_p_	5.32	16	T_p_	T_p_
#18 (F98 IDH2-R172K)	0.00	n/a	F_n_	5.65	7	T_p_	T_p_	4.12	20	T_p_	0.00	999	F_n_	F_n_
#19 (F98 IDH2-R172K)	0.81	23	U	2.22	8	T_p_	T_p_	5.81	9	T_p_	3.29	18	T_p_	T_p_
*Correct detection*			*13 (68%)*			*15 (79%)*	*17 (89%)*			*7 (37%)*			*9 (47%)*	*12 (63%)*
*Incorrect detection*			*2 (11%)*			*0 (0%)*	*2 (11%)*			*8 (42%)*			*1 (5%)*	*7 (37%)*
*Uncertain case (CRLB >20%)*			*4 (21%)*			*4 (21%)*	*0 (0%)*			*4 (21%)*			*9 (47%)*	*0 (0%)*

*2HG/water cut-off for 2HG absent voxel = 0.20;

^#^2HG/water cut-off for 2HG absent voxel = 0.52

For the FID–truncation+MRUI, there were 10 correctly detected (true negative) and 2 uncertain cases out of 12 in Group A. There were 3 correctly detected (true positive), 2 incorrect (false negative), and 2 uncertain cases out of 7 in Group B. The overall percentage of correct detection (either true positive or true negative) of 2HG with this method was ~68% (13/19). For the voxel–specific baseline+MRUI, there were 9 correctly detected and 3 uncertain cases in Group A. There were 6 correctly detected and 1 uncertain cases in Group B. Thus, by incorporating the voxel–specifically measured baselines into the quantitative analysis, the overall percentage of correct detection of 2HG was improved to ~79% (15/19). In particular, the sensitivity (correct detection for 2HG–present voxels) was greatly improved from ~43% (3/7) to ~86% (6/7), and the total number of incorrect detection was reduced from 2 to none.

For the simulated baseline+LCModel, there was no correctly detected case with 8 incorrect (false positive) and 4 uncertain cases out of 12 in Group A. On the other hand, 2HG was correctly detected for all 7 voxels in Group B. The overall percentage of correct detection of 2HG with this method was ~37% (7/19). For the voxel–specific baseline+LCModel, there were 4 correctly detected and 8 uncertain cases in Group A. There were 5 correctly detected cases with 1 incorrect and 1 uncertain cases in Group B. The overall percentage of correct detection of 2HG was improved to ~47% (9/19). Importantly, the total number of incorrect detection was drastically reduced from 8 to 1 by incorporating the measured baselines.

### Addressing uncertain cases with CRLB > 20% by defining cutoff 2HG_MRS_

In order to deal with those uncertain cases with CRLB>20% in the results obtained with measured baselines, which took considerable portions (4/19 for MRUI and 9/19 for LCModel), particularly for Group A, cutoff 2HG_MRS_ values for each of the MRUI and the LCModel data were defined based on the relationships between 2HG_LC–MS_ and 2HG_MRS_. The linear regression resulted in 2HG_MRS_ = (0.96 × 2HG_LC–MS_ (×10^−5^)) + 0.06 for MRUI (r = 0.79, p<0.001) and 2HG_MRS_ = (1.06 × 2HG_LC–MS_ (×10^−5^)) + 0.36 for LCModel (r = 0.81, p = 0.008).

By converting the highest 2HG_LC–MS_ in Group A into 2HG_MRS_, the resulting cutoff 2HG_MRS_ for 2HG–absent voxels were 0.20 and 0.52 for MRUI and LCModel, respectively. Using these cutoff values, for the voxel–specific baseline+MRUI, there were 10 correctly detected cases out of 12 in Group A. All 7 cases were correctly detected in Group B. The overall percentage of correct detection of 2HG was further improved to ~89% (17/19). For the voxel–specific baseline+LCModel, there were 6 correctly detected cases out of 12 in Group A. Six out of 7 cases were correctly detected in Group B. The overall percentage of correct detection of 2HG was improved to ~63% (12/19). Thus, compared to those results without measured baselines, the overall percentages of correct detection were improved from ~68% to ~89% for MRUI, and from ~37% to ~63% for LCModel. In particular, the sensitivity of MRUI was improved from ~43% to 100%, and the specificity of LCModel was improved from 0% to 50%. The resulting mean 2HG_MRS_ for the correctly detected cases in Group B were 3.97±2.56 for MRUI and 4.80±1.15 for LCModel.

## Discussion

In this report we have investigated in animal models of brain tumors at 9.4T the potential impact of incorporating voxel–specifically measured baselines into the basis set on the noninvasive detection of 2HG by using short TE 1H–MRS in combination with MRUI and LCModel. To address 2HG_MRS_ with CRLB>20%, as an illustration, we have defined cutoff 2HG_MRS_ values for 2HG–absent voxels.

We used F98 glioma cells transfected with human IDH1/2 gene–cloned lentiviral vectors and implanted in rat brain. F98 anaplastic glioma clone is classified as a malignant brain tumor, and known to show an infiltrative growth pattern analogous to that of human GBM [28]. The overexpression of IDH1/2 mutations and overproduction of 2HG in our animal models were clearly shown.

The main resonance of 2HG signal resides in ~2.0–2.5 ppm where signals from at least 4 other metabolites (GABA, Glu, Gln, and NAAG) [9, 10] also contribute. The severe spectral overlap is exacerbated by the presence of MMs—the primary component of spectral baseline, and lipid in the case of fat infiltration that is often accompanied in patients with brain tumors [47, 48]. For this reason, the majority of those previous studies have employed spectral editing methods for the detection of 2HG with relatively long TEs [9, 10] either for effective suppression of background metabolite signal [10] by taking advantage of different J–evolution of different coupled spin systems [13] or as a consequence of using a pair of spectral editing pulses with a relatively long pulse duration in order to achieve high spectral selectivity [9, 10]. Given the strong dependence of the accuracy of metabolite quantification on spectral baseline [18–20] and its relatively short T2 [21], such long TEs employed in spectral editing methods are clearly beneficial on the one hand [10, 49, 50]. On the other hand, as discussed by the authors [10, 14], the resulting 2HG content may be prone to quantitative errors resulting from substantial signal loss due to relaxation. In addition, the signal loss arising from both relaxation and, for coupled spins, J–evolution of spins can also render precise quantification of metabolites other than 2HG challenging in the edited spectra, which may also be important for better understanding of the pathogenesis and progression of brain tumors with gene mutations and for the development of novel therapeutic strategies [15–17]. For instance, the reduction of Glu levels resulting from the decreased activity of the branched–chain amino acid transaminase 1 (BCAT1) enzyme has been suggested to be strongly associated with the pathogenesis of the IDH1 mutated gliomas [15, 51]. As well, the down–regulation of glutathione (GSH) in IDH1 mutated gliomas was suggested as a potential therapeutic target [16]. Therefore, together with the fact that short TE 1H–MRS is most commonly used in metabolite quantification without the need of pulse sequence programming and sequence optimization procedure, improvement of the performance of such a conventional short TE approach is important. To this end, the previous reports that among MR–visible metabolites Glu and Gln are most influenced by baseline [18, 19, 22], and the fact that the 2HG signal directly overlaps with Glu and Gln, both dictate the investigation of the potential impact of voxel–specifically measured baselines on the 2HG detection.

To address this issue, first, for the comparison purpose, 2HG was quantified without measured baselines by using FID–truncation in combination with MRUI (FID–truncation+MRUI) and by using the current version of LCModel with built–in MM and lipid modelling (simulated baseline+LCModel). Using these methods, our results corroborate even at 9.4T those previously reported technical challenges in the detection of 2HG at short TE [9, 12, 14]. In our study, excellent specificity but with relatively low sensitivity was obtained for the FID–truncation+MRUI. For the simulated baseline+LCModel, excellent sensitivity was obtained but with no true negative case. Such relatively low specificity and high sensitivity with LCModel in the detection of 2HG has also been reported in a recent human study [12]. The FID–truncation employed in our study is simple to perform and frequently used for the removal of MM signal when measured baselines are not available, and its performance has been reported previously [23, 35, 39, 40]. Therefore, while it results in SNR reduction in the spectra to a certain extent particularly for those metabolites with relatively short T2, the FID–truncation method in combination with MRUI is a reasonable approach for the investigation of the impact of measured baselines on 2HG detection. For LCModel, such FID–truncation can also be performed. However, the current version of LCModel used in our study already employs an extended basis set including modeled MMs and lipids [45, 47, 48, 52], and therefore does not require a prior knowledge about baselines.

By incorporating voxel–specifically measured baselines, the overall performance of both MRUI and LCModel were improved. In particular, the total numbers of incorrect detection were substantially reduced for both MRUI (from 2 to none) and LCModel (from 8 to 1) prior to addressing those uncertain cases with CRLB>20%. In clinical practice, uncertain or indeterminate diagnostic cases can occur, for which a follow–up examination is usually performed. Thus, false diagnosis is far more problematic than uncertain cases. In this regard, the drastic reduction of incorrect detection of 2HG by incorporating measured baselines in our study clearly demonstrates the advantage of this approach. While the retrieved SNR of the spectra with respect to that with FID–truncation in the case of MRUI needs to be accounted for to a certain extent, the improved 2HG detection by using measured baselines reconfirms the strong dependence of quantitative outcome on the baseline as previously reported [18–20]. The improved performance, in turn, also indicates the difficulty of modeling MMs and lipids due to their variability in vivo. The current version of LCModel has model spectra for MMs and lipids, and to avoid over–parameterization of the analysis several spectral components were combined into a single component (e.g., MM resonances at 1.95, 2.08 and 2.25 ppm combined into a single component (i.e., ‘MM20’), and lipid resonances at 2.04, 2.25 and 2.8 ppm combined into ‘Lip20’) followed by a soft constraint imposed on the ratios between lipid components and between MM components (i.e., ‘Concentration Ratio Priors’) [45, 47]. By this sophisticated modeling, LCModel is known to better separate between MMs and lipids [45]. However, for instance, the proportion or relative intensity of the lipid resonances can differ for different lipid composition [47, 53]. As well, while in LCModel the residual of fit even after using the currently implemented modeled MM and lipid baselines is supposed to be accounted for by the calculated spline function, these combined strategies may not be as good in performance as voxel–specifically measured baselines, particularly at high field [20, 23].

Even with the measured baselines, the performance, esp., the specificity, with both MRUI and LCModel requires further improvement, which again demonstrates the challenges of 2HG detection at short TE even at 9.4T. This was mainly due to the presence of those uncertain cases (CRLB>20%). Such uncertain cases are frequently encountered [9, 10, 12, 14], which most likely result from the limited SNR and spectral dispersion of 1H–MRS spectra. In the cases with CRLB>20%, previous studies implicitly regarded those relatively low 2HG concentrations as 2HG–absent [14], and those relatively high 2HG concentrations as 2HG–present [12] with no defined cutoff value. For an effectively isolated 2HG peak from both background metabolites and spectral baselines as in the cases with spectral editing methods [9, 10], at least a threshold concentration of 2HG for reliable detection can be determined by using simulation or in phantom for a given SNR of spectra [9]. However, at short TE, such preliminary analysis is difficult due to the severe spectral overlaps of 2HG with background metabolites and spectral baselines, the concentrations of both of which can vary in vivo. For this reason, we have resorted, as an illustration, to the relationships between the 2HG_LC–MS_ and the correctly detected 2HG_MRS_, given that the Group A and Group B were stratified based on the 2HG_LC–MS_ values. Using this approach, the overall percentages of correct detection of 2HG were further improved. In particular, the sensitivity of MRUI was improved from ~43% to 100%, and the specificity of LCModel was improved from 0% to 50%. Such a cutoff 2HG_MRS_ for 2HG–absent voxels may also be defined, for instance, from the mean 2HG_LC–MS_ between the highest 2HG_LC–MS_ in Group A and the lowest 2HG_LC–MS_ in Group B, instead of from the highest 2HG_LC–MS_ in Group A alone. However, in our study, those uncertain cases occurred far more often in Group A, for which 2HG_MRS_ values should tend to be small. Therefore, the cutoff 2HG_MRS_ converted from the highest 2HG_LC–MS_ in Group A should be more reasonable. Although not attempted in our study due to the small number of samples, such a cutoff 2HG_MRS_ may be better defined, for instance, empirically from a preliminary study, in which case cutoff 2HG_MRS_ values should be determined in conjunction with both SNR and linewidth of spectra that are known to simultaneously influence fitting precision [22]. This means that such a cutoff value needs to be determined specific to a given experimental setting including imaging parameters and the choice of a data post–processing software. Once a cutoff 2HG_MRS_ is defined retrospectively from a preliminary study, uncertain cases may then be prospectively addressed in the following studies.

Previous studies reported comparable performance of time–domain and frequency–domain analyses in metabolite quantification [25–27]. Although in our study such a direct comparison between the two approaches is difficult due to the small sample size, substantial improvement in the detection of 2HG by employing voxel–specifically measured baselines was seen for both. The resulting 2HG_MRS_ values from LCModel tend to be higher than those from MRUI. Such an overestimation of frequency–domain quantification relative to time–domain approach has also been reported previously [27].

Our study has limitations. Overall, the improved performance of the short TE MRS approach with the proposed method has been demonstrated. However, the current results presented in our study does not strongly support its applications directly to the diagnosis of each individual patients. Prior to clinical applications, the proposed method should be validated in a larger number of animals and animal groups with different 2HG content, and more importantly in human patients with resulting sensitivity and specificity acceptable to radiologists and neurosurgeons. Although there were strong correlations between 2HG_LC–MS_ and 2HG_MRS_ in our study, the measurement errors can also result from biological variability. For this reason, we have excluded in the final data analysis those samples with intermediate 2HG_LC–MS_, and focused our analysis on the detection rather than quantification of the onco–metabolite with only two animal groups of 2HG–absent and 2HG–present. The highest 2HG_LC–MS_ in the former was more than 20 times lower than the lowest 2HG_LC–MS_ in the latter. Metabolite–nulled spectra estimated by using an inversion recovery technique are prone to residual metabolite signal [20, 23]. To minimize such contamination of baseline spectra, however, we have employed a double inversion technique [23, 34, 35]. The baseline spectra resulting from double inversion are known to be subject to T1–weighting and low signal yield [36]. Increasing the number of signal averages to retrieve the signal would require a prohibitively long scan time. For this reason, the metabolite–nulled spectra were apodized in our study as previously reported [37], which also facilitates the smoothness of baseline that is commonly assumed in the baseline modeling [45]. Our metabolite–nulled spectra for CN VOIs are in close agreement with those previously reported using rat brain at the same field strength [20]. In our study, a calculated metabolite basis set was used instead of those measured in phantom. However, the performance of a calculated basis set was shown to be equivalent to that of a measured basis set [23, 54]. Therefore, the detection errors observed in our study is not likely due to inaccurate spectral bases, but a consequence of the limited performance of 1H–MRS. For improved 2HG detection at short TE, we proposed incorporating measured baselines instead of using the built–in, modeled baselines currently employed in LCModel. One of the disadvantages with the proposed approach would be the difficulty of lipid quantification, which may also be important for glioma research, given that lipid is associated with glioblastomas and its status after treatment [47, 48]. At high field more detailed baseline information is known to be required due to higher spectral dispersion [20, 23]. However, it should be noted that with currently available hardware setting at 3.0T a better shimming and a comparable or even higher SNR than those obtained in our study can be attained. Specifically, whereas the mean linewidth of water was ~15 Hz for the mean voxel volume of ~9.6 mm^3^ in our study, a linewidth of as narrow as ~6 Hz was reported to be achievable at 3.0T for a voxel volume of 8 cm^3^ [10] or even larger [9]. In addition, while our data were acquired by using a single channel receiver coil, 8–32 channel receiver coils are used in clinical scanners [9, 10, 12]. As both SNR and spectral dispersion are coupled to influence the quantitative precision [22], the incorporation of voxel–specifically measured baselines is expected to improve 2HG detection at clinical field strength as well, and in this context our results at 9.4T can be informative for upcoming clinical studies. Finally, voxel–specific acquisition of metabolite–nulled spectra requires additional scan time. Thus, the total scan time required for a short TE spectrum and a baseline can be comparable to that required for J–difference editing [9, 10]. For the latter, 2HG can be detected in the J–difference (subtracted) spectrum, and quantification of other metabolites can also be performed from the reference spectrum (without editing pulses), but with the quantitative outcome potentially prone to error for all metabolites due to relaxation. The long TE single shot method [10] can directly detect 2HG with only half the scan time required for both J–difference editing and the short TE acquisition with baseline measurement. Therefore, the extra scan time can be dedicated for an additional spectrum at short TE. For such a long TE single shot method with an extra short TE spectrum, only 2HG is potentially subject to quantitative error due to relaxation. Upon validation in human studies of the proposed approach with short TE acquisition in combination with baseline measurement, the different pros and cons of these three different approaches for 2HG detection should be taken together in the study design.

## Conclusions

In vivo detection of 2HG by using the conventional short TE approach is challenging even at 9.4T. While the proposed method should be validated in a larger number of animals and more importantly in human patients, the incorporation of voxel–specifically measured spectral baselines into the spectral basis set may potentially improve its performance.

## Supporting Information

S1 File The 2HG quantification by LC-MS. (XLSX)

S2 File The 2HG quantification by MRS. (XLSX)

S3 File The linear regression between 2HG_LC-MS_ and 2HG_MRS_. (XLSX)
